# Case report: Thoughts on two cases of total anomalous pulmonary venous connection complicated with pulmonary artery hypertension

**DOI:** 10.3389/fcvm.2023.1075168

**Published:** 2023-01-26

**Authors:** Ling Yan, Yaxin Zhou, Dayan Li, Lingli Li, Hong Tang

**Affiliations:** ^1^Department of Cardiology, West China Hospital, Sichuan University, Chengdu, China; ^2^Rehabilitation Medicine Center, West China Hospital, Sichuan University, Chengdu, China

**Keywords:** total anomalous pulmonary venous connection, pulmonary artery hypertension, echocardiography, congenital heart disease, cardiac surgery

## Abstract

The two primary pathological alterations of total anomalous pulmonary venous connection (TAPVC), a rare cyanotic congenital heart disease (CHD), are right heart failure and pulmonary artery hypertension (PAH). The timing and prognosis of surgery depend on the level of pulmonary hypertension. Surgery will not be an option after Eisenmenger syndrome appears. In light of this, it is crucial to assess patients’ PAH. In order to aid in the following treatment of related types of diseases, this article studied and compared the echocardiographic features and disease development of one adult and one child TAPVC patients complicated with PAH.

## Introduction

Congenital heart disease with pulmonary artery hypertension (CHD-PAH) is the most prevalent form of the first category of pulmonary hypertension and is characterized by elevated pulmonary artery pressure due to a body-lung shunt (1). According to a European study, 5–10% of adult patients with CHD had PAH, and the prevalence of CHD-PAH in China is 3–4 times higher than that in Western nations (2). Due to PAH, many patients with CHD miss out on the chance to have surgery, raising their risk of heart failure and mortality. Right heart catheterization is the gold standard test for diagnosing PAH, while echocardiography is integral for screening and longitudinal evaluation of PAH and can be used for differential etiological diagnosis and cardiac function estimation simultaneously.

Total anomalous pulmonary venous connection (TAPVC) is a rare cyanotic CHD that accounts for 0.4–1.5% of CHDs (3, 4). In TAPVC, all pulmonary veins are connected to the right atrium instead of the left atrium, either directly or *via* the systemic venous system, allowing oxygenated blood to continuously flow into the pulmonary circulation. According to the drainage site of the pulmonary veins, TAPVC can be classified as infracardiac, supracardiac, intracardiac, or mixed type. About 5–10% of them are mixed type (5), whose clinical characteristic is the presence of two or more anomalous pulmonary venous connection sites. Large atrial septal defects (ASDs) are common complications in patients with TAPVC, and the right-to-left shunt allows oxygenated blood to enter the systemic circulation to meet physiologic requirements. However, excessive pulmonary blood flow from the anomalous pulmonary veins might still easily cause right cardiac overload and pulmonary hypertension. The 1-year mortality rate with TAPVC is up to 80%, (6) and adult presentation of TAPVC is rare. In order to aid in the following treatment of related types of diseases, this article studied and compared the echocardiographic features and the disease development of one adult and one child TAPVC patients complicated with PAH.

## Case report

### Case 1

**FIGURE 1:**
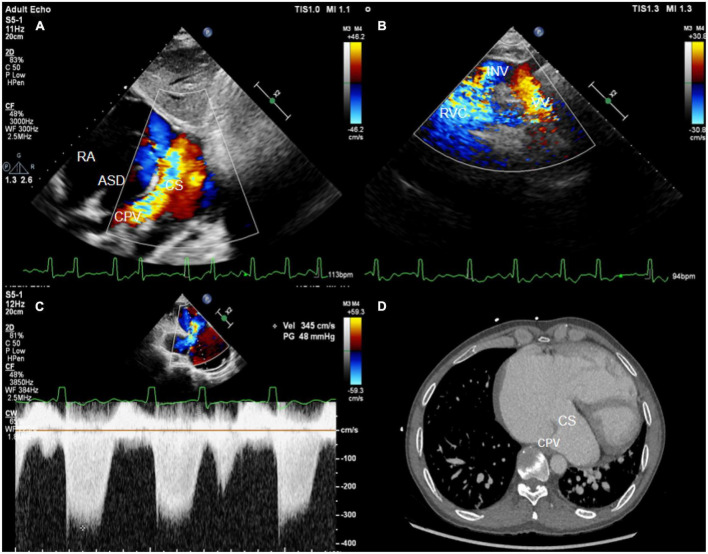
**(A)** The subxiphoid biatrial view showed the common pulmonary venous lumen draining into the coronary sinus. **(B)** The suprasternal fossa section showed a vertical vein flowing into the innominate vein. **(C)** The spectrum of tricuspid regurgitation. **(D)** The CT image showed common venous trunk opening into the coronary sinus. CPV, common pulmonary venous lumen; RA, right atrium; ASD, atrial septal defect; VV, vertical vein; INV, innominate vein; CS, coronary sinus; RVC, right superior vena cava.

The 47-year-old male patient felt chest discomfort after activities for 6 months and started experiencing palpitations and shortness of breath. There was no similar symptom before, and the patient can engage in general physical activity. At the first visit, the patient was noted to have cyanosis by physical examination, along with a grade 3/6 pan-systolic murmur over the left sternal edge, peripheral oxygen saturation of 78%, blood pressure of 95/68 mmHg, heart rate of 84 bpm, and slight edema in both lower limbs. A complete right bundle branch block was visible on the electrocardiogram. Echocardiography showed that the right pulmonary vein and the left inferior pulmonary vein formed a venous trunk that drained into the right atrium through the coronary sinus, and the left superior pulmonary vein was combined with the innominate vein through the vertical vein and into the right atrium (Figures 1A, B). The right atrium and right ventricle were also enlarged, and the pulmonary artery was widening. The size of the ASD was approximately 36 × 46 mm (Figure 1C). Then, the diagnosis of ASD with TAPVC (mixed type) and pulmonary hypertension was performed by echocardiography and CT (Figure 1D). ASD repair and TAPVC repair were carried out in June 2020, and the patient recovered well from surgery.

### Case 2

**FIGURE 2:**
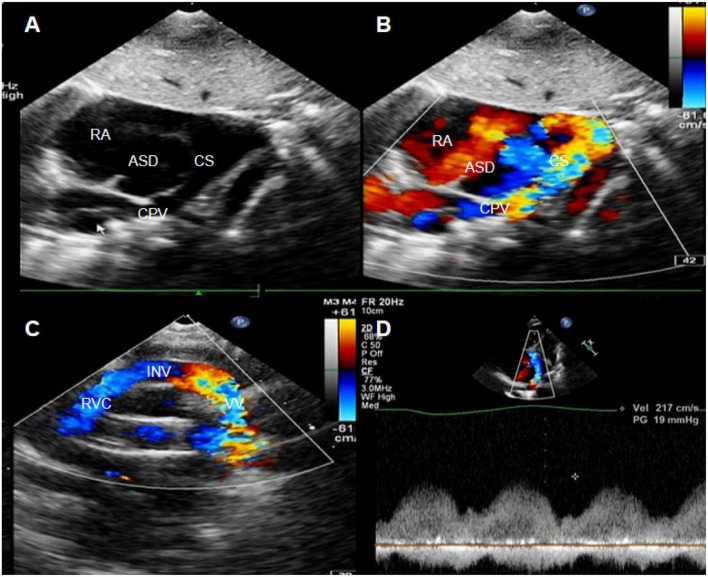
**(A,B)** The subxiphoid biatrial view showed the common pulmonary venous lumen draining into the coronary sinus. **(C)** The suprasternal fossa section showed a vertical vein flowing into the innominate vein. **(D)** The blood flow spectrum of coronary sinus stenosis. Red arrows indicate the stenosis of the coronary sinus.

A 1-year-old female newborn was hospitalized due to worsening cyanosis. A patent foramen ovale and an ASD (7 mm) with TAPVC (mixed type) were detected (Figure 2C). Similar to case 1, the pulmonary vein drained into the right atrium through the coronary vein and the vertical vein. The diameter of the coronary sinus was about 6 mm (Figures 2A, B), and the blood flow was accelerated by about 2.17 cm/s at the entry point into the right atrium (Figure 2D). Meanwhile, severe pulmonary hypertension was also diagnosed *via* the tricuspid regurgitation pressure gradient and right heart catheterization. Then, the surgery was performed, and the patient had no discomfort throughout the 3-year follow-up.

## Discussion

Pulmonary artery hypertension is the most common complication in patients with CHD. Almost all patients with TAPVC have PAH (7), and the cause of PAH is different from other types of CHD. In cardiac TAPVC, pulmonary venous confluence drains directly into the right atrium. Meanwhile, the blood flow in the systemic circulation returns to the right atrium and then enters the pulmonary system, leading to increased pulmonary blood flow. The upshot is increased pressure in the right heart due to excessive pulmonary blood flow and unchanged pulmonary vascular bed. ASD can allow a part of the right atrial blood to flow into the left atrium to participate in the systemic circulation. The larger the defect is and the greater the shunt blood flow is, the lesser the blood flow into the pulmonary circulation is and the lower the pulmonary circulation pressure will be. Hence, the diameter of the defect is related to pulmonary artery pressure. Another major cause of PAH in patients with TAPVC is obstruction of pulmonary venous return (8). The suction effect caused by normal contraction and relaxation of the left ventricular is the main factor to ensure the return of the pulmonary vein to the left atrium, due to anomalous pulmonary drainage to the right atrium, the loss of left ventricular pumping caused by pulmonary venous reflux disorder, elevated pulmonary venous pressure, and hence increased pulmonary artery pressure in patients with TAPVC. Furthermore, a considerable proportion of patients with TAPVC has pulmonary venous trunk obstruction (9), which aggravates pulmonary venous hypertension as well as PAH.

The two instances discussed here both had ASD with mixed TAPVC. However, the patient in case 1 can live better over 40 years, while the patient in case 2 experiences cyanosis in 1 year. The size of the ASD and whether or not the pulmonary venous return is obstructed are directly correlated with the clinical symptoms of TAPVC. Patients are more prone to develop right heart failure and pulmonary hypertension symptoms earlier when the ASD is modest and the pulmonary venous return is blocked. For instance, in case 2, the atrial septal shunt was small, and PAH progressed to a severe stage rapidly. The development of PAH, however, was delayed and the degree was modest when the ASD was large, and there was no evident venous return blockage, as in case 1, who experienced right heart failure symptoms at the age of 47 years and had moderate levels of pulmonary hypertension.

Correct preoperative diagnosis and accurate anatomical description are essential for the surgical treatment of such patients (10). Diagnostic uncertainty is related to the highly variable anatomy of mixed TAPVC. At least 15 mixed variants of different forms have been found. Chowdhury et al. (10) divided mixed TAPVC into three types, namely, the “2 by 2” pattern, the “3 by 1” pattern, and the “bizarre” pattern. In most cases, echocardiography is sufficient and desirable, but sometimes only two or three veins can be identified, resulting in misdiagnosis. The characteristics of echocardiography in the diagnosis of TAPVC are as follows (11). (1) The common pulmonary vein cavity is seen behind the left atrium and there is no pulmonary vein opening in the left atrium. (2) Obvious pulmonary hypertension and right heart enlargement can be detected. (3) All surviving patients have a right-to-left shunt. The two examples in this article are the mixed TAPVC “3 + 1” type. It is simple to overlook one of them because of the varied position of pulmonary venous drainage (12). The main causes are the lack of a comprehensive and standard scan sequence, which ignores the scan of alternative ectopic drainage routes like the scan of the suprasternal fossa with or without vertical veins, and the content with detecting the common pulmonary venous cavity drainage channel.

## Conclusion

In general, severe PAH develops in infancy in the majority of patients with TAPVC. Only surgical intervention can save patients’ lives, and whether they have pulmonary venous return obstruction has a significant impact on their prognosis. The timing and prognosis of surgery depend on the level of pulmonary hypertension, so it is crucial to assess patients’ PAH.

## Data availability statement

The original contributions presented in this study are included in this article/supplementary material, further inquiries can be directed to the corresponding author.

## Ethics statement

The studies involving human participants were reviewed and approved by the Ethics Committee of West China Hospital, Sichuan University. Written informed consent to participate in this study was provided by the participants’ legal guardian/next of kin. Written informed consent was obtained from the individual(s), and minor(s)’ legal guardian/next of kin, for the publication of any potentially identifiable images or data included in this article.

## Author contributions

All authors listed have made a substantial, direct, and intellectual contribution to the work, and approved it for publication.
